# Thermal tolerance of giant salmonfly nymphs (*Pteronarcys californica*) varies across populations in a regulated river

**DOI:** 10.1093/conphys/coae043

**Published:** 2024-07-05

**Authors:** Christine E Verhille, Michael MacDonald, Ben Noble, Gavin Demorest, Alzada Roche, Kayleigh Frazier, Lindsey K Albertson

**Affiliations:** Department of Ecology, Montana State University, Bozeman, MT 59717, USA; Department of Ecology, Montana State University, Bozeman, MT 59717, USA; Department of Ecology, Montana State University, Bozeman, MT 59717, USA; Department of Ecology, Montana State University, Bozeman, MT 59717, USA; Department of Ecology, Montana State University, Bozeman, MT 59717, USA; Department of Ecology, Montana State University, Bozeman, MT 59717, USA; Department of Ecology, Montana State University, Bozeman, MT 59717, USA

**Keywords:** aerobic scope, aquatic invertebrates, stonefly, thermal habitat, thermal optima

## Abstract

Warming of aquatic ecosystems is transforming the distribution, phenology and growth of the organisms dependent upon these ecosystems. Aquatic insects such as stoneflies are especially vulnerable to warming because the aquatic nymph stage of their life cycle depends on cool, well-oxygenated, flowing water habitat. We tracked thermal effects on available aerobic capacity of the aquatic nymph stage of an iconic and vulnerable stonefly species, the giant salmonfly (*Pteronarcys californica*), to compare habitat thermal regime measurements for two salmonfly populations from habitats separated by a gradient in summer weekly maximum temperatures. Contrary to expectations, the thermal optima range of the warmer habitat population was cooler than for the cooler habitat population. We posit that this unexpected interpopulation variation in thermal response is more strongly driven by diel and seasonal thermal variability than by the highest summer temperatures experienced within respective habitats. Additionally, we show that summer daily maximum temperatures could result in periodic limits in available aerobic capacity to support work of the warmer habitat nymphs and may be the mechanism underlying reduced abundance relative to the upstream cooler habitat population. Our findings provide insight into potential thermal and metabolic mechanisms that could regulate the success of ecological and culturally important aquatic insect species experiencing global change. We conclude that thermal regimes and thermal variation, not just mean and maximum temperatures, are critical drivers of aquatic insect responses to water temperatures.

## Teaser

Contrary to our prediction, salmonfly (Pteronarcys californica) nymphs originating from the warm extreme of a thermal gradient had a cooler metabolism-based optimal temperature range than nymphs originating from a cooler habitat. We posit that these local adjustments reflected the combination of thermal extremes and thermal variation experienced within the habitats.

## Introduction

Warming of aquatic ecosystems caused by global climate change, damming and land use alteration is transforming the distribution, phenology and growth of the organisms dependent upon these ecosystems. These changes are especially problematic in Western United States and Canada, where aquatic ecosystems are dependent on melting mountain snowpack for water replenishment (Pederson *et al.*, 2011). Shifts to earlier and more variable seasonal snowmelt runoff have resulted in earlier and more variable transitions to low summer base flows in Rocky Mountain West streams (Pederson *et al.*, 2011). Longer periods of summer base flows combined with warmer summer air temperatures (Pederson *et al.*, 2011) have resulted in warming summer and fall temperatures of most northwestern US rivers (Isaak *et al.*, 2012, 2017), including habitats of cold-water salmonids and their macroinvertebrate food sources (Isaak *et al.*, 2018). Although the physiological mechanisms underlying cold-water fish responses to warming habitat temperatures are well studied, the physiology of important macroinvertebrate food sources of these fish has received less attention.

**Figure 1:**
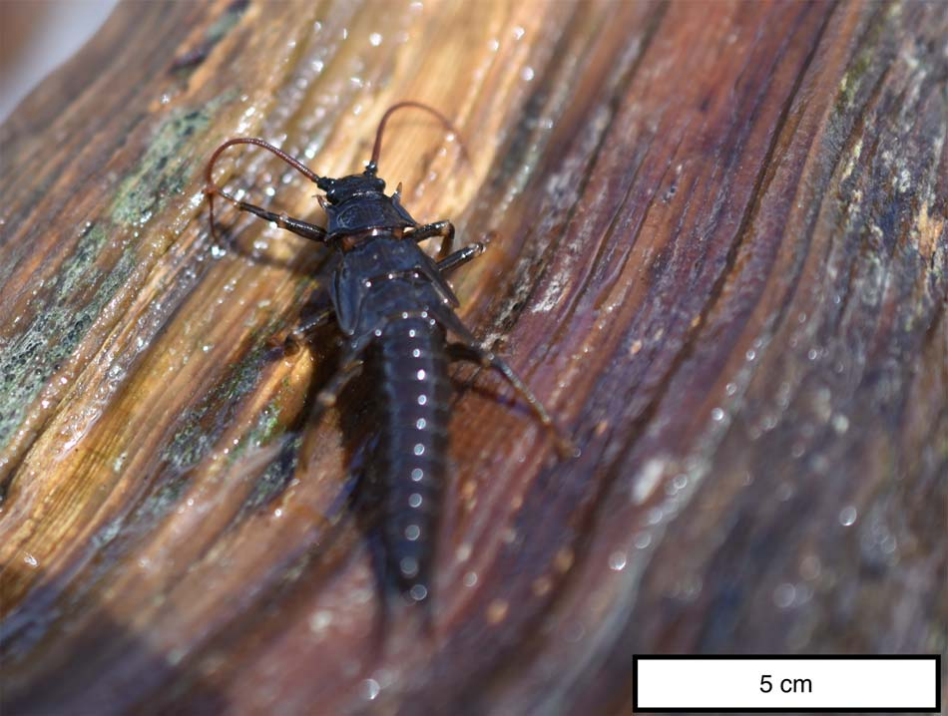
Salmonfly (*P. californica*) nymph captured on the Madison River, MT, USA. This nymph is estimated to be in at least the second-year age class. Photograph taken by M. MacDonald.

The river stonefly (*Pteronarcys californica*, also known, and here onwards referred to, as the salmonfly; Fig. 1) is a large-bodied (in some cases exceeding 6 cm) aquatic insect of the order Plecoptera that depends on cool, well-oxygenated, flowing water habitat (Freilich, 1991). Because of their large size, salmonflies serve as an important nutrient source for aquatic and terrestrial consumers and control organic matter processing (Ruesink and Srivastava, 2001; Lecerf and Richardson, 2011; Walters *et al.*, 2018). This iconic macroinvertebrate is distributed throughout rivers draining the Coast, Cascade, Rocky and Sierra Nevada mountain ranges of North America (Townsend and Pritchard, 1998). The first 2–5 years of the salmonfly life history is an aquatic larval nymph life stage (Freilich, 1991; DeWalt and Stewart, 1995; Townsend and Pritchard, 1998). After many molts during the aquatic larval life stage, a salmonfly emerges as a terrestrial adult, which soon reproduces and dies (Gregory *et al.*, 2000; Walters *et al.*, 2018). Salmonfly emergence timing is synchronized among individuals to enhance reproductive success, resulting in an immense nutrient input for aquatic and terrestrial consumers (Rockwell and Newell, 2009; Wesner *et al.*, 2019) and an attractant to recreational anglers (Stagliano, 2010).

Salmonfly populations show substantial declines throughout their contemporary range (Elder and Gaufin, 1973; Vinson, 2008; Stagliano, 2010; Nehring *et al.*, 2011; Birrell and Nelson, 2018; Anderson *et al.*, 2019a). These declines are thought to be a result of anthropogenic modifications to aquatic habitat structure and flow (Freilich, 1991), excess fine sediment (Kowalski and Richer, 2020) and warming summer water temperatures (Anderson *et al.*, 2019a). Indeed, captive salmonfly nymph growth rates decline at temperatures above 15°C (Townsend and Pritchard, 2001) and salmonflies are currently rare in habitats where maximum weekly August temperature exceeds 18.6°C (Huff *et al.*, 2006; Anderson *et al.*, 2019a). Specifically, factors associated with regulated rivers are hypothesized to strongly influence salmonfly populations. Recent work on the dammed Madison River in southwestern Montana has revealed that salmonfly emergence and density are tightly coupled to water temperatures (Stagliano, 2010; Anderson *et al.*, 2019a, 2019b). Salmonflies are functionally extinct from ~50% of their historical range in the Madison River below the most downstream dam and are projected to continue an upstream range contraction due to temperature limitations into the future (Anderson *et al.*, 2019a).

Warming water temperatures can result in a progression of non-lethal effects that can be characterized using metabolic thermal response curves. Resting and maximum metabolic rate responses to warming temperature can be tracked to determine the aerobic scope (determined as the difference between resting and maximum metabolic rate) thermal response. This approach has been applied to characterize sublethal thermal effects for a broad taxonomic range of aquatic ectotherms (Pörtner and Farrell, 2008); however, only a small number of studies have applied this approach to aquatic insects (see Verberk *et al.*, 2016 for review). As an estimate of the available capacity to perform metabolic work above and beyond basic maintenance requirements, aerobic scope can be applied as a proxy for the effects of temperature on the capacity of an organism to carry out the non-maintenance tasks necessary for successful survival and reproduction (e.g. digesting a meal, growing, molting and emerging as adults) in nature. Previous studies on thermal tolerance of the stonefly *Dinocras cephalotes* (Verberk *et al.*, 2013) and salmonflies (Frakes *et al.*, 2021) showed reduced thermal tolerance in hypoxic relative to normoxic conditions, suggesting that thermal limits of these species are oxygen dependent. Therefore, aerobic scope measurements could be meaningful to investigate optimal temperatures and non-lethal effects of warming on salmonfly nymphs.

Here, we track salmonfly nymph metabolic responses to warming water to investigate the variation in metabolic thermal responses among two populations originating from habitats separated by a thermal gradient. Based on previous population-level responses observed on the same river (Anderson *et al.*, 2019a, 2019b) and local adaptation within the closely related perlid stoneflies, specifically *Hesperoperla pacifica*, to thermal regimes (Shah *et al.*, 2021), we hypothesize that warming water temperatures will limit the aerobic metabolic capacity of salmonfly nymphs and acclimation or adaptation to local habitat conditions will result in variable responses between the different salmonfly populations that reflect the thermal gradient separating them. We predict that nymphs will possess low aerobic scope at low and high temperatures and high aerobic scope at intermediate temperatures. Due to local adjustments of the two experimental populations, we predict that the range of water temperatures where aerobic scope is maximal will reflect the thermal regimes experienced in the respective habitats, with nymphs captured in the cooler habitat possessing maximum available aerobic scope at cooler water temperatures than the nymphs captured in the downstream warmer habitat.

## Materials and Methods

### Study system, sites and environmental conditions

**Figure 2:**
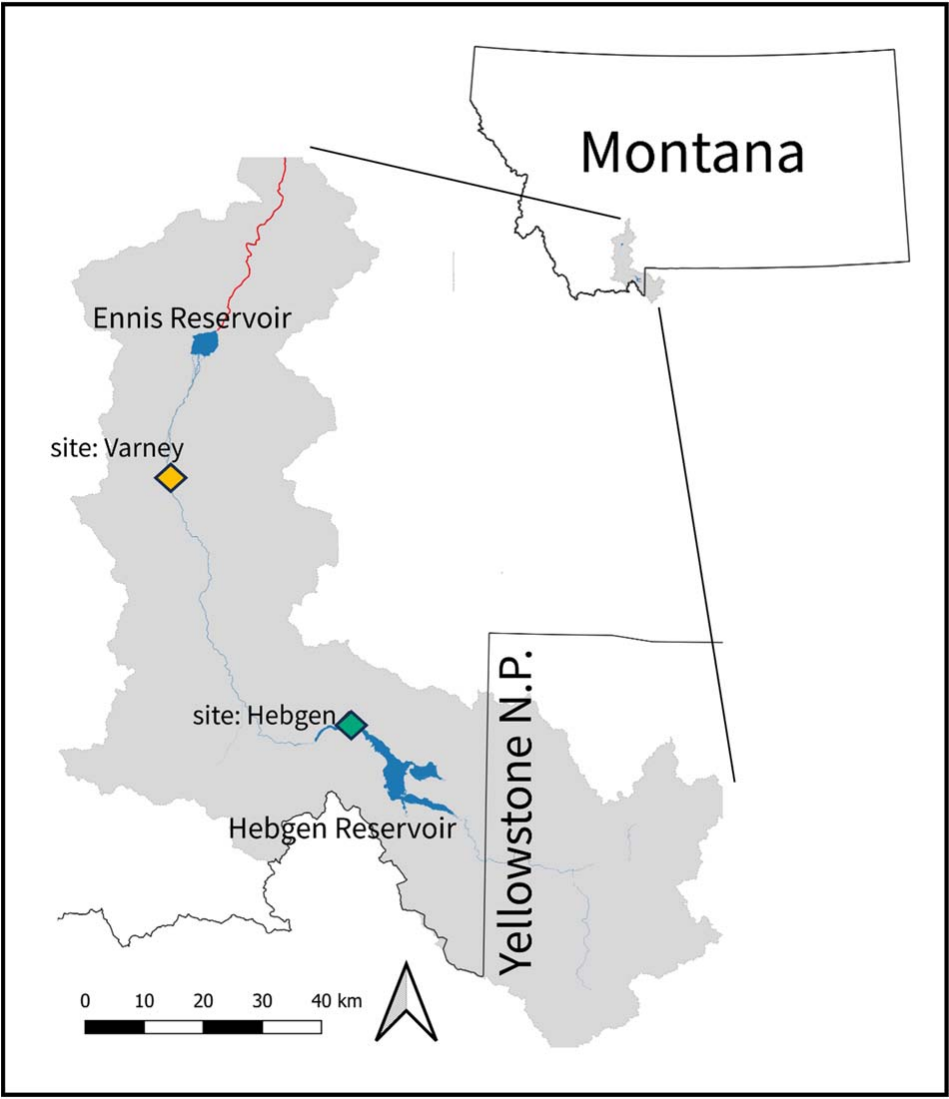
Study area within the Madison River. Salmonfly (*P. californica*) nymphs were collected from Hebgen (green) and Varney (orange) sites, which are located between two major reservoirs. Red line downstream of Ennis Reservoir indicates river reach where thermal conditions are no longer suitable for salmonflies.

Salmonfly nymphs were collected from the Madison River, in southwestern Montana, USA (44.92°N, −111.61°W) from mid-August to mid-September in 2020 and 2021. The Madison River originates in northwestern Yellowstone National Park and flows northwesterly through broad valleys for ~210 km until its confluence with the Jefferson and Gallatin Rivers where it forms the Missouri River at Three Forks, MT. Within the Upper Madison River (Fig. 2), two dams are operated by Northwestern Energy for water storage and hydroelectric production from Hebgen and Ennis Reservoirs. For this study, nymphs were collected from two sites within the stretch of river separating Hebgen Reservoir from Ennis Reservoir.

The habitat sites for salmonfly population sampling were identified based on prior research documenting population-level responses to the Madison River thermal gradient (Anderson *et al.*, 2019a, 2019b). The Hebgen site (hereafter Hebgen; 44.86°N, 111.35°W) is located immediately downstream of Hebgen Reservoir, in the cool, relatively stable hypolimnetic dam discharge, and supports densities of salmonflies averaging 117 m^−2^ (Fig. 2; Table 1). The Varney Bridge site (hereafter Varney; 45.23°N, 111.75°W) is ~73 river kilometres (RKM) downstream of the Hebgen site. The RKM is the distance measurement including the river path between points as opposed to the straight line connecting those points.

**Table 1:** Salmonfly nymph densities at Varney Bridge and Hebgen Dam reported in our previously published work (Anderson *et al.*, 2019a) and number of days habitat temperature daily mean, minimum (Min.) and maximum (Max.) exceeded 18.6°C, which is the August weekly maximum temperature our previous research observed nymph occurrence to be rare (Anderson *et al.*, 2019a, 2019b)

	Varney		Hebgen	
	2020	2021	2020	2021
Nymph density (m^−2^)	84.3		116.7	
Hours >18.6°C	165	146	17	4
# Days				
Mean >18.6°C	2	7	0	0
Min. >18.6°C	0	0	0	0
Max. >18.6°C	25	17	2	2

Summer Madison River water temperatures gradually warm from upstream to downstream, with the warmest water temperatures occurring at the most downstream site (Varney). Varney can achieve mean summer weekly maximum temperatures that are 2.4°C greater than at Hebgen and this corresponds with a 32.4-m^−2^ decrease in nymph density relative to at Hebgen, averaging 84 m^−2^ (Anderson *et al.*, 2019a; Fig. 2; Table 1). Although salmonfly nymph distribution extended downstream of Ennis Reservoir as recently as 1977 (Anderson *et al.*, 2019a), the rise in temperature of water passing through Ennis Reservoir creates contemporary thermal conditions that are unsuitable for salmonflies, resulting in a severely diminished salmonfly population at all locations in the Madison River downstream of Ennis Reservoir, making the Varney site nearly the downstream extent of present-day Madison River salmonfly population distribution. We hypothesized a physiological mechanism underlying these previously reported declining abundance trends across the thermal gradient to be increased maintenance metabolic demands and decreased capacity to metabolically support essential survival tasks above and beyond basic maintenance with warming habitat.

**Figure 3:**
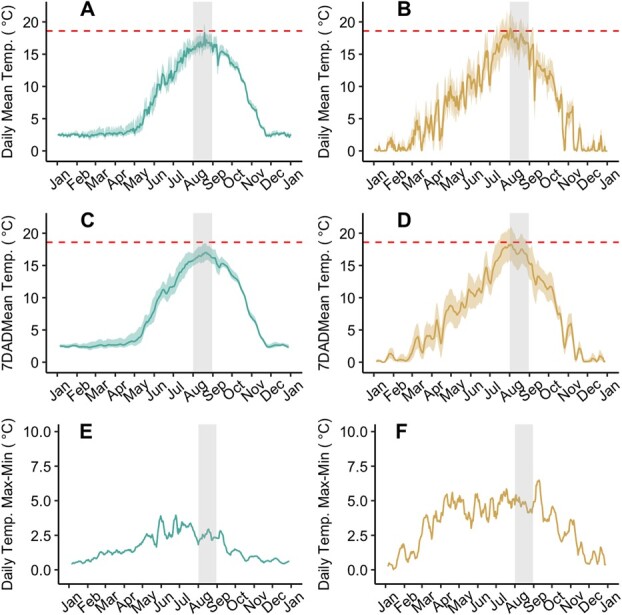
Temperature data logged in 2020 from two sites [Hebgen (green) in A, C, E and Varney Bridge (orange) in B, D, F] within the Madison River, MT where salmonfly (*P. californica*) nymphs were collected for assessment of thermal responses for metabolism (see Supplementary Fig. S3 for 2021 temperature logs). (A, B) Daily mean (solid line) and maximum and minimum (respectively shaded above and below the solid line) temperatures; (C, D) sliding average of the 7-day average daily mean [7DADMean; (solid line)], maximum (7DADMax) and minimum (7DADMin) (respectively shaded above and below the solid line) temperatures; and (E, F) diel variation (Daily Max-Min) calculated daily as the daily minimum recorded temperature subtracted by the daily maximum recorded temperature. Grey rectangles overlay August measurements. Salmonflies are relatively rare where August mean weekly maximum temperature exceeds 18.6°C (Huff *et al.*, 2008; Anderson *et al.*, 2019a), indicated by red dashed line in A–D.

We continuously recorded water temperature at Hebgen and Varney at 1-hour intervals from fall 2019 to fall 2021. Water temperature was logged using HOBO Pro V2 temperature loggers (HOBO U23 Pro V2, Onset MA USA) anchored as close as possible to the river thalweg. We computed various aspects of the thermal regime for either the month of August (Figs 3 and Supplementary Fig. S3; Tables 1 and 2) or from January to October (Figs 3 and Supplementary Fig.S3; Table 3) to characterize thermal differences between the Hebgen and Varney sites during the summer of 2020 and 2021. We also calculated the number of days during the winter where mean, minimum and maximum water temperature fell to ≤0°C for each site using logger data from November 2019 to April 2020 and November 2020 to April 2021.

**Table 2:** Summer environmental conditions at the Varney and Hebgen sites on the Madison River, MT where salmonfly (*Pteronarcys californica*) nymphs were collected for assessment of thermal responses for metabolism. All metrics are based on thermograph logger data recorded in August of 2020 and 2021. Temperatures of 11.4 and 19.4°C for Varney habitat and 17.6 and 24.6°C for Hebgen habitat were focused on in temperature metrics as the cool and warm limits of the optimal temperature ranges based on aerobic scope reported by this study. Max.: maximum; Min.: Minimum; Temp.: Temperature; 7DADMax: sliding average of the average of seven consecutive daily maximum temperatures across the specified time period; CDD: cumulative degree days; CDH: cumulative degree hours; TSM_7DADMax: Thermal safety margin based on the August 7DADMax; TSM_Max: Thermal safety margin based on the maximum observed August temperature; Daily Max-Min: diel variation in temperature

	Year	
	2020	2021
Varney		
August Max. (°C)	21.9	21.8
August mean 7DADMax (°C)	19.4	18.6
August CDD (°C)	529	508
August CDH (°C)	12 692	12 186
August Daily Max-Min (°C)	10.6	10.0
TSM_7DADMax (°C)	0	0.8
TSM_Max (°C)	−2.5	−2.4
August # Days		
Mean Temp. 11.4–19.4°C	31	31
Min. Temp. 11.4–19.4°C	30	31
Max. Temp. 11.4–19.4°C	13	20
Mean Temp. <11.4°C	0	0
Min. Temp. <11.4°C	1	0
Max. Temp. <11.4°C	0	0
Mean Temp. >19.4°C	0	0
Min. Temp. >19.4°C	0	0
Max. Temp. >19.4°C	18	11
Hebgen		
August Max.	19.9	18.7
August mean 7DADMax (°C)	17.9	17.7
August CDD (°C)	511	513
August CDH (°C)	12 263	12 315
August Daily Max-Min (°C)	5.4	4.8
TSM_7DADMax (°C)	6.7	6.9
TSM_Max (°C)	4.7	5.9
August # days		
Mean Temp. 17.6–24.6°C	1	2
Min. Temp. 17.6–24.6°C	0	0
Max. Temp. 17.6–24.6°C	25	17
Mean Temp. <17.6°C	30	29
Min. Temp. <17.6°C	31	31
Max. Temp. <17.6°C	6	13
Mean Temp. >24.6°C	0	0
Min. Temp. >24.6°C	0	0
Max. Temp. >24.6°C	0	0

**Table 3:** Winter temperature metrics for the Varney and Hebgen sites. The winter season was defined as 11 November to 16 April, for both 2019 to 2020 and 2020 to 2021. Min.: minimum; Max. maximum

	Varney		Hebgen	
	2019–20	2020–21	2019–20	2020–21
February Min. (°C)	−0.03	−0.06	1.5	1.8
Winter # days				
Min. < 0°C	88	105	0	0
Max. < 0°C	10	19	0	0

Salmonfly nymphs of the penultimate instar stage were collected by kick netting and immediately transported to the laboratory for measurements. Upon capture, nymphs of the target life stage were identified based on body size and wing pad development (Townsend and Pritchard, 1998). Nymphs of the penultimate instar stage undergo the final molt in the following spring and emerge as reproductive adults early in the summer (Townsend and Pritchard, 1998). Nymphs from each site were transported to the lab and briefly habituated to lab conditions at water temperatures identical to those at capture. Nymphs captured from the river were immediately placed in a 20-L bucket filled with aerated river water for transport back to the laboratory (~1.5 hours). At the laboratory, nymphs were maintained in a 20-L holding tank until metabolic rate measurements were performed. The holding tank water was aerated using an aquarium air pump (Tetra Whisper10, Blacksburg, VA) and recirculated through a chiller (Model Isotemp4100, Fisher Scientific, Waltham, MA), which maintained temperatures within ±0.1°C. All water used to hold nymphs was Madison River water collected from the capture site. Salmonfly nymphs were held for a maximum duration of 172 hours and a minimum duration of 2.5 hours (1.5-hour transport +1-hour habituation to the experimental setup when adjustments to test temperature was not required) before the start of metabolic rate trials.

### Metabolism measurement protocol

Resting (RMR) and maximum (MMR) metabolic rates of individual nymphs were measured at temperatures ranging from ~7.8°C to 30.1°C at ~1°C increments, with each nymph tested at only one temperature. In total, we measured the metabolic rate on 64 nymphs from Hebgen (30 in 2020 and 34 in 2021) and 50 nymphs from Varney (26 in 2020 and 24 in 2021). The larger number of nymphs from Hebgen than Varney reflects the higher than anticipated thermal optima for Hebgen nymphs and interindividual variation in metabolic rates around the thermal optima range for both populations. The number of nymphs measured per 1°C temperature increment ranged from one to nine. Measurements were discarded for seven Hebgen and six Varney nymphs due to either equipment malfunctions or excessive activity overnight during measurements for RMR.

Metabolic rate measurements were conducted from 12 August to 9 September in 2020 and 20 August to 15 September in 2021 on solitary nymphs, each at a single temperature. Salmonfly nymphs were moved from the holding tank to respirometer chambers before the start of an experimental trial. The temperature of the respirometer chamber was initially set to the temperature of the holding tank, then slowly increased or decreased to the test temperature at a rate of 2°C h^−1^ (Verhille *et al.*, 2016).

Replicate measurements to estimate RMR were performed overnight via intermittent respirometry (Steffensen, 1989; Cech Jr, 1990; Chabot *et al.*, 2016; Svendsen *et al.*, 2016) in custom-designed respirometers. We continuously recorded O_2_ uptake overnight from ~7 p.m. to ~9 a.m., while nymphs remained undisturbed in the respirometer chambers and chambers were alternated between a 15-minute seal to track the rate of metabolic removal of oxygen and a 3-minute flush with fresh bath water to restore chamber water oxygen levels. The flush seal timing allowed for ~46 replicate seals overnight per nymph. The seal duration was timed to prevent respirometer water dissolved oxygen (DO) saturation from falling below 90%, and the pumps were calibrated to deliver 625 ml of fresh water to the respirometer chamber during the flush to replenish water DO saturation to nearly 100% and flush out accumulated nitrogenous waste (Svendsen *et al.*, 2016). The flush seal cycle was automated using a timer-controlled pump.

Measurements to estimate MMR were performed during the next morning also via intermittent respirometry concurrent with a 36-minute chase protocol which began at ~9 a.m. This protocol involved continuous recording of O_2_ uptake while chasing and flipping the nymph onto its back using a pipe cleaning brush within the respirometer chamber. At the beginning of the chase protocol, the respirometer system was set to flush with fresh bath water and then nymphs were chased continuously for two full flush seal cycles, allowing for two estimates of MMR during the chase protocol. If nymphs lost the ability to right themselves (loss of righting response) before the end of the 36-minute chase protocol, chasing was ceased, but O_2_ uptake measurements and the flush seal cycle continued, and data were retained.

Background water respiration rates were measured before each nymph was placed into a respirometer and again after the nymph was removed the following day.

Immediately after completing MMR measurements on an individual nymph, the nymph was measured for dry mass. Nymphs were desiccated in a tabletop drying oven set to 60°C for 2–3 days until weight stabilized. Dry mass of the nymph was recorded as the final stabilized weight after desiccation.

### Respirometers

A single respirometer consisted of a 150-ml Erlenmeyer flask (129–137-ml end respirometer water volume) sealed with a rubber stopper. Water within the respirometer was circulated with a 2-cm magnetic stir bar. To protect the nymphs from the stir bar, a plastic mesh screen floor was elevated 2 cm from the base of the respirometer. Each respirometer was held in a bath of temperature-controlled, aerated, river water that also served as a freshwater bath supply to the respirometer. Four holes were drilled through the rubber stopper for insertion of an oxygen optode, temperature probe, inflow tubing to flush the respirometer chamber with fresh bath water and a pipe brush to agitate salmonfly nymphs during MMR measurements. The hole in the rubber stopper for the pipe brush was just wide enough to allow sufficient agitation of the larvae while also acting as an overflow port while flushing the respirometer.

The respirometer bath was supplied with a continuous flow of collection site river water from two 80-L temperature-controlled water reservoirs. Reservoir water was maintained at test temperatures using a one-fourth horsepower inline water chiller (Model DS-3 Aqua Logic Delta Star, USA) and a 0.5-kW Cygnet titanium aquatic heater (Aqua Logic, USA). Water was aerated by pumping through a PVC vertical aeration column using a high-volume sump pump (Supreme® Aqua-Mag 9.5b, Danner MFG, Islandia, NY, USA). Water also continuously passed through a QStar ultraviolet sterilizer (ALQ25IL, Aqua Logic, USA) to minimize accumulation of microbiota.

Temperature-compensated water oxygen saturation in each respirometer was monitored continuously using a fibre-optic oxygen optode (PyroScience, Model OXROB10, Germany) and a submersible temperature probe (PyroScience, Model TSUB21, Germany) connected to an optical oxygen and temperature meter (PyroScience, Model FirestingO2, Germany) and associated software. We calibrated each oxygen probe weekly following two-point calibrations protocols using 100% air-saturated water (aerated distilled water) and 0% air-saturated water (150-ml distilled water with 2-g dissolved Na_2_SO_3_).

### Analyses

All statistical analyses were performed in the statistical computing environment R (R Core Team, 2021).

Metabolic rates were calculated as slope of oxygen removal over time using lm() in base R. As oxygen was tracked as DO saturation (%), slopes were converted from DO saturation (%)/min to units of milligrams per minute based on oxygen solubility and respirometer volume. Metabolic rates were not adjusted for background respiration because we did not detect background respiration at any of the test temperatures.

The RMR of an individual nymph was estimated as the average of the lower 10% quantile of replicate overnight measurements. The respirometry system was set to perform ~46 seals overnight, and most seals allowed for two separate slope measurements resulting in, on average, 70 ± 28 (mean ± 1 SD) RMR measurements per individual nymph. To estimate the RMR of an individual nymph, we pooled all overnight slope measurements with an *R^2^* > 0.90 (Svendsen *et al.*, 2016) for that individual and calculated an average of the lower 10% quantile of the remaining measurements (Chabot *et al.*, 2016). Therefore, only one RMR estimate per individual nymph was calculated and each individual was measured at only one temperature.

The MMR estimate for an individual nymph was determined as the highest measurement meeting the *R^2^* > 0.90 threshold during the MMR protocol (Svendsen *et al.*, 2016). Although the respirometry system was set to perform two seals during the chase protocol, most seals allowed for five separate slope measurements resulting in, on average, 10 ± 1 (mean ± SD) metabolic rate measurements per individual nymph to estimate MMR from. As for RMR measurements, each nymph was measured for MMR at only one test temperature.

Aerobic capacity was quantified as aerobic scope (AS) and factorial aerobic scope (FAS). The AS was calculated by subtracting RMR from MMR for each nymph separately. The FAS was calculated by dividing RMR into MMR for each nymph separately.

We modelled the salmonfly nymph metabolic variables: RMR, MMR, AS and FAS as a function of temperature using non-linear fitting. The best-fit model formula and associated starting coefficients for non-linear least squares model parameterization was determined, based on Akaike information criterion (AIC) scores, separately for each metabolic variable and population using the fitModels() function of the thermPref package (mdjbru-R-packages, 2023). The best-fit model formula and starting coefficients were than applied to the nlsLM() function of the minpack.lm package (Elzhov *et al.*, 2016) to fit the model. When the AIC of multiple models outputted from fitmodels() or the nlsLM() functions fell within 2 units of each other, the simplest competitive model was chosen as the best fit. The amount of variation in the relationship between the metabolic variable and temperature explained by the best fit model was estimated as the adjusted *r*^2^ of the linear regression between the observed metabolic variable values and model fitted values at the same temperature. Using the finalized best fit MMR, AS and FAS models, separately for each population, we then used the predict() function to estimate the temperature where each of these metabolic variables peaked (Topt) and the temperature range peak was maintained across (Topt range). The Topt range was estimated based on maintenance of 90% (Topt90) and 80% (Topt80) of the peak AS.

Allometric effects of variation in salmonfly nymph mass on metabolism have not been investigated, and our dataset did not allow for quantification of mass effects on metabolism. Therefore, we explored effects of allometric coefficients ranging from 0.00 to 1.00 applied to RMR and MMR measurements on the AS of individual nymphs (Supplementary Table S1). Thermal tolerance ranges determined based on the metabolic thermal responses only slightly varied with applied allometric coefficient, and the population differences in thermal tolerance range were not impacted by this variation (Supplementary Table S1; Figs S1, S2, and S4). As a result, metabolic rate calculations were not corrected for mass.

August and winter habitat thermal regimes were summarized from the temperature loggers deployed in the field using numerous metrics to explore correspondences between inter-population variation in metabolic thermal response and habitat thermal regimes. Daily, hourly, monthly and aggregate metrics were considered to explore ecologically meaningful aspects of the thermal regime to salmonfly nymphs and help explain patterns we observed in the laboratory experiment (Turschwell *et al.*, 2016). Cumulative degree days (CDDs) were calculated as the number of days multiplied by the sum of the mean temperature of each day. Cumulative degree hours (CDHs) were calculated similarly to CDDs, except hourly temperatures were summed and multiplied by the total number of hours. Both CDD and CDH were quantified over specified periods of time (e.g. for the month of August). The 7-day average daily mean (7DADMean), maximum (7DADMax) and minimum (7DADMin) temperatures were calculated as the sliding average of seven consecutive daily mean, maximum or minimum temperatures across the specified time period (e.g. for the month of August). The diel variation in temperature (Daily Max-Min) was calculated as the daily maximum recorded temperature minus the minimum temperature and averaged across the specified time period.

Thermal safety margins (TSMs) of Hebgen and Varney salmonfly nymph populations for the summers of 2020 and 2021 were estimated based on the upper limit of the Topt90 temperature range for each population and year separately. Two iterations of TSM were calculated for each population within the population-specific habitat by subtracting one of two estimates of the maximum habitat temperature from the upper limit of the Topt90 range (Deutsch *et al.*, 2008). The two estimates of maximum habitat temperature included the August hourly maximum temperature (TSM_Max) and the August 7DADMax (TSM_7DADMax).

## Results

As a result of equipment failures or high levels of nymph activity during RMR measurements, metabolic rate measurements failed for 11 Hebgen nymphs (3 in 2020 and 8 in 2021) and 6 Varney nymphs (4 in 2020 and 2 in 2021), resulting in a final count of 64 Hebgen nymph measurements (27 in 2020 and 26 in 2021) and 44 Varney nymph measurements (22 in 2020 and 2021). Dry mass of nymphs ranged from 0.034 to 0.311 g. The two populations were similar with Hebgen nymphs averaging 0.133 g (min: 0.034 g; max: 0.311 g) and Varney nymphs averaging 0.142 g (min: 0.046 g; max: 0.302 g).

### Metabolism

**Figure 4:**
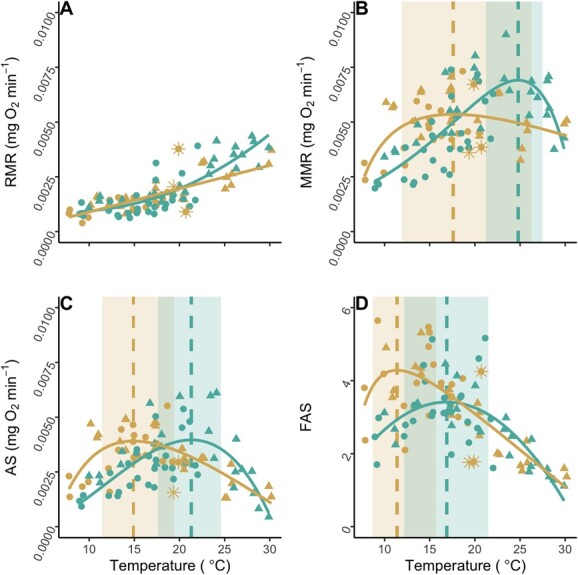
Metabolic rate and scope thermal responses for salmonfly (*P. californica*) nymphs from the cooler, less variable Hebgen (green) and warmer, more variable Varney (orange) sites on the Madison River, MT collected in 2020 (circles) and 2021 (triangles). Resting metabolic rate (RMR; A), maximum metabolic rate (MMR; B), absolute aerobic scope (AS; C) and factorial aerobic scope (FAS; D) for *n* = 53, and *n* = 44 salmonflies from Hebgen (27 in 2020 and 26 in 2021) and Varney (22 in 2020 and 2021), respectively. Asterisk symbols represent observations where salmonfly could not right itself or flee during MMR measurements. Solid lines depict fitted values based on the model characterizing the population-specific relationship between the metabolic variable and temperature (see Table 4 for models and statistical coefficients). The thermal optima temperature and range (temperature range 90% peak was maintained) for MMR, AS and FAS estimated from the models are indicated by vertical lines and rectangles, respectively.

The RMR was modelled as an exponential relationship for the cooler Hebgen site nymphs and a linear relationship for the warmer Varney site nymphs (Table 4; Fig. 4A). For Hebgen nymphs, the RMR increased from 8.95 × 10^−4^ mgO_2_min^−1^ at 8°C to 3.83 × 10^−3^ mgO_2_min^−1^ at 28°C. In contrast, the RMR of Varney nymphs ranged from 6.81 × 10^−4^ mgO_2_min^−1^ to 2.81 × 10^−3^ mgO_2_min^−1^ across the same temperatures. Therefore, the RMR models described a 4.3-fold increase in RMR for Hebgen salmonfly nymphs and a 4.1-fold increase in RMR for Varney salmonfly nymphs between 8 and 28°C.

**Table 4:** Equations for best-fit models predicting metabolic rates and scopes at temperature for salmonfly (*P. californica*) nymphs from the Varney and Hebgen sites on the Madison River, MT. Values for model coefficients are listed below each model. RMR: resting metabolic rate; MMR: maximum metabolic rate; AS: aerobic scope; FAS: factorial aerobic scope; X: temperature; df: degrees of freedom; *r*^2^: adjusted *r*^2^ of the linear model of fitted values versus observed values; RSE: residual standard error.

Dependent variable	Population	Model	RSE	df	*r* ^2^
RMR	Varney	a + b × X	0.00053	42	0.54
		a = −0.0001697			
		b = 0.0001064			
	Hebgen	a + b × X^1.5^ + c × X^2^	0.00060	48	0.71
		a = 0.001060934			
		b = −0.0000371			
		c = 0.0000105			
MMR	Varney	a + b × log(X)^2^ + c × log(X) + d × log(X)/X	0.00109	40	0.26
		a = −0.179513194			
		b = −0.009180676			
		c = 0.077496005			
		d = 0.233963066			
	Hebgen	exp(rho×X)-exp(rho×tmax-(tmax -X)/delta)	0.00129	52	0.54
		rho = 0.149283294			
		tmax = 31.49806582			
		delta = 6.697500268			
AS	Varney	a + b × log(X)^2^ + c × log(X) + d × log(X)/X	0.00083	40	0.47
		a = 0.035180899			
		b = −0.003037786			
		c = 0.003954095			
		d = −0.109130843			
	Hebgen	a + b × X^2^ × log(X) + c × X^3^	0.00105	48	0.44
		a = −0.000219669			
		b = 0.0000134			
		c = −0.00000149			
FAS	Varney	a + b × log(X)^2^ + c × log(X) + d × log(X)/X	0.8106	40	0.53
		a = −270.5207792			
		b = −12.35124834			
		c = 108.9238294			
		d = 388.232928			
	Hebgen	a + b × X + c × X^2^	0.6946	48	0.48
		a = −1.142947682			
		b = 0.539868645			
		c = −0.0159921			

The MMR was modelled as polynomial relationships for both Hebgen and Varney nymphs (Table 4; Fig. 4B). For Hebgen nymphs, MMR was 2.03 × 10^−3^ mgO_2_min^−1^ at 8°C, peaked at 7.10 × 10^−3^ mgO_2_min^−1^ at 24.8°C then fell to 5.98 × 10^−3^ mgO_2_min^−1^ at 28°C. Hebgen nymphs, on average, maintained 90% of the peak MMR from 21.2 to 27.5°C, whereas for Varney nymphs, the MMR was 2.75 × 10^−3^ mgO_2_min^−1^ at 8°C, peaked at 5.35 × 10^−3^ mgO_2_min^−1^ at 17.6°C, then fell to 4.62 × 10^−3^ mgO_2_min^−1^ at 28°C. Varney nymphs, on average, maintained 90% of the peak MMR from 11.9 to 26.3°C.

The AS was modelled as a polynomial relationship for Hebgen and Varney nymphs (Table 4, Fig. 4C). For Hebgen nymphs, the AS was 0.80 × 10^−3^ mgO_2_min^−1^ at 8°C, peaked at 3.96 × 10^−3^ mgO_2_min^−1^ at Topt of 21.3°C, then fell to 2.04 × 10^−3^ mgO_2_min^−1^, which is 52% of peak AS, at 28°C. Hebgen nymphs, on average, maintained 90% of the peak AS from 17.6 to 24.6°C. For Varney nymphs, the AS was 1.90 × 10^−3^ mgO_2_min^−1^ at 8°C, peaked at 3.91 × 10^−3^ mgO_2_min^−1^ at a Topt of 14.9°C, then fell to 1.64 × 10^−3^ mgO_2_min^−1^, which is 42% of peak AS, at 28°C. Varney nymphs, on average, maintained 90% of the peak AS from 11.4 to 19.4°C.

The FAS was modelled as a polynomial relationship for Hebgen and Varney nymphs (Table 4, Fig. 4D). For Hebgen nymphs, the FAS was 2.2 at 8°C, peaked at 3.4 at 16.9°C, then fell to 1.4, which is 41% of peak FAS, at 28°C. Hebgen nymphs, on average, maintained 90% of the peak FAS from 12.2 to 21.5°C. For Varney nymphs, the FAS was 3.5 at 8°C, peaked at 4.3 at 11.4°C, then fell to 1.5, which is 35% of peak FAS, at 28°C. Varney nymphs, on average, maintained 90% of the peak FAS from 8.7 to 15.7°C.

### Loss of responsiveness

Three of the nymphs from the warmer Varney site became unresponsive to stimuli at 19.4, 19.9 and 20.7°C during the chase protocol (Fig. 4). Despite loss of righting response (LRR) in these individuals, RMR and MMR were measured and retained. Nymphs from the cooler Hebgen site never displayed LRR, regardless of test temperature.

### Habitat temperatures

Field temperature measurements showed August temperatures at Hebgen to be cooler and less variable than at Varney (Fig. 3; Tables 1 and 2). The August maximum measured temperature was ~18°C at Hebgen and 22°C at Varney, and August mean 7DADMax was ~18°C for Hebgen and 19°C for Varney in 2020 and 2021. August diel variation in temperature (i.e. the average of the daily difference between maximum and minimum measured temperature) at Hebgen (~5°C) was nearly half that of Varney (~10°C). Seasonal variation in temperature was also less for Hebgen than for the Varney site (Table 3). The maximum August temperature and minimum February temperature measured at Hebgen through 2020 and 2021 were 19.9 and 1.6°C, which is an 18.3°C difference. At the Varney site, the maximum August temperature and minimum February temperature measured through 2020 and 2021 were 21.9 and −0.1°C, which is a 21.8°C difference.

Measurements of August habitat temperatures (Table 2) of the cooler Hebgen site tended to be cooler than the Hebgen nymph Topt90 range lower limit (17.6–24.6°C). Mean daily habitat temperature was within the Topt90 range for only 1–2 days or 92–100 hours in August in 2020 and 2021. Most of the time, habitat temperature was outside of the nymph Topt90 range, and it was lower than the Topt90 range lower limit. Measured daily minimum temperature fell below the Topt90 range lower limit for 31 days or 642–644 hours in August. Daily maximum measured temperature never exceeded the Hebgen nymph Topt90 range upper limit and fell within the Topt90 range for 17–25 of the days in August. Comparing August Hebgen habitat temperatures to the Hebgen nymph Topt80 range (15.9–25.8°C), mean daily habitat temperature fell within the Topt80 range for 26–27 days of August and fell below the Topt80 range lower limit for 4–5 days. Temperatures measured at Hebgen in August never exceeded the Topt80 range upper limit. August Hebgen habitat temperatures fell within the Topt90 range for ~1700 CDH and within the Topt80 range for ~9000 CDH.

August habitat temperatures measured at the warmer Varney site (Table 2) tended to fall within the Topt90 range (11.4–19.4°C) for Varney nymphs with frequent periods of temperatures exceeding the Topt90 range upper limit. The August mean daily temperature measured at the Varney habitat fell within the Varney nymph Topt90 range for 31 of the 31 days or 648–658 of 744 hours, depending on year. Most of the time Varney habitat temperature was outside of the nymph Topt90 range, it exceeded the Topt90 range upper limit. The maximum daily temperature exceeded the Varney nymph Topt90 range upper limit for 11–18 of the 31 days in August or 86–93 of the 744 hours in August. This resulted in an average of 1817 CDH above the Varney nymph Topt90 range upper limit in August. Varney August habitat daily minimum temperature fell below the Varney nymph Topt90 range lower limit for only 3 hours during 1 day. Comparing August Varney habitat temperatures to the Varney nymph Topt80 range (10.1–21.6°C), mean daily habitat temperature fell within the Topt80 range for all 31 days of August and never fell below the Topt80 range lower limit. Maximum daily temperature exceeded the Topt80 range upper limit for 1–3 days (3–6 hours) depending on the year. August Varney habitat temperatures fell within the Topt90 range for ~10 000 CDH and within the Topt80 range for ~12 000 CDH.

The thermal safety margins were greater than or equal to 5°C for nymphs from the cooler Hebgen and small to negative for nymphs from the warmer Varney site (Table 2). Based on Topt90 range upper limits and the measured 7DADMax habitat temperatures, the TSM of Hebgen nymphs was 6.7–6.9°C, compared to 0.0–0.8°C for Varney nymphs (TSM_7DADMax), depending on the year. The TSM based on the maximum measured temperature at the Hebgen site (TSM_Max) was 4.7 and 5.9°C compared to −2.5 to −2.4°C at the Varney site.

Based on field temperature measurements, the winter season was defined as from 11 November to 16 April of the following year. This was based on the first autumn date, and last spring date sub-freezing temperatures were observed at the Varney site (sub-freezing temperatures were never observed at the Hebgen site). The lowest minimum daily temperature occurring at Hebgen during the winter season was 1.5–1.8°C, depending on the year. During the winter season, Varney habitat minimum temperature fell to ≤0°C for a total of 88–105 days, depending on year (Table 3). For 10 of these sub-zero days at the Varney site, maximum temperature also did not exceed 0°C.

## Discussion

We compared metabolic thermal responses of penultimate instar salmonfly nymphs belonging to populations originating from habitats separated by a thermal gradient and found surprising trends that suggest complex influences of habitat thermal regimes including diel and seasonal variation on salmonfly nymph thermal tolerance. Contrary to our prediction that the cooler site population (Hebgen) would possess a cooler thermal optimal range for AS relative to the warmer site population (Varney), we observed higher optimal temperatures in the cooler site population. Although salmonflies at Varney experienced the warmest maximum temperatures, we posit that the mechanisms underlying their lower thermal optimal range may be related to the greater diel and seasonal thermal variation experienced within the Varney relative to Hebgen habitat. These findings illustrate that despite decades of research on aquatic ectotherm responses to temperature (e.g. Fry, 1971; Verberk *et al.*, 2016; Cuenca Cambronero *et al.*, 2018; Lefevre *et al.*, 2021), the specific aspects of environmental spatial and temporal variation driving organismal physiological responses leading to population-level effects are still not fully understood. Additionally, the effects of temperature extremes and variation on organisms can vary with organism behaviour and life stage (Morash *et al.*, 2021; Kefford *et al.*, 2022; Seebacher *et al.*, 2023). Our results underpin the challenges of understanding and predicting temperature effects on organisms in natural ecosystems and show that when considering organismal responses to natural thermal regimes, thermal variation is as important as thermal extremes or means.

This study applied a novel approach to quantify physiological responses of wild-captured penultimate instar salmonfly nymphs while minimizing acclimatory responses to captive conditions. Before metabolism measurements were performed, nymphs were held for no longer than 7 days in capture-site water maintained at capture temperature. The advantage of this approach is it limits acclimatory responses to captive conditions that could result in adjustments to nymph thermal responses. A disadvantage of this approach is the lack of control for post-prandial elevation of RMR, but previous work on stonefly species found no effects of starvation on RMR (Nagell, 1973). Therefore, we expect the trends reported here reflect actual metabolic thermal responses of penultimate instar salmonfly nymphs in their natural habitat.

To compare metabolic thermal response curves of nymphs originating from the warm and cool habitat, we described trends of RMR, MMR, AS and FAS responses to warming. The RMR, or metabolic demands of basic organismal maintenance, of both Hebgen and Varney nymphs increased with warming and at a faster rate for Hebgen than Varney nymphs (Fig. 4A). The MMR thermal response was hump-shaped for both Hebgen and Varney nymphs, with a peak at warmer temperature for Hebgen nymphs (24.8°C) than Varney nymphs (17.6°C) (Fig. 4B). These findings suggest that, although maintenance metabolic demands increase more quickly with warming for Hebgen, relative to Varney nymphs, Varney nymphs lose maximum aerobic metabolism at lower temperatures than the cooler-habitat Hebgen nymphs. The AS thermal response was hump-shaped for both Hebgen and Varney nymphs, but with a peak (Topt) at a warmer temperature for the cooler-habitat Hebgen nymphs. Based on maintenance of 90% of peak AS, we concluded that the thermal optima range (Topt90 range) was 17.6–24.6°C for Hebgen nymphs and 11.4–19.4°C for Varney nymphs (Fig. 4C). Notably, whereas no Hebgen nymphs exhibited LRR, regardless of test temperature, 3 out of 15 Varney nymphs held overnight at temperatures ≥19.4°C exhibited LRR, supporting 19.4°C as an upper limit for at least a portion of the Varney nymph population. Therefore, contrary to our prediction, the Topt range for scope available to support non-maintenance aerobic metabolic work for the cooler-habitat Hebgen nymphs was higher than the Topt range of the warmer-habitat Varney nymphs. As far as we are aware, this is the first quantification of the thermal response of salmonfly nymph AS.

Comparisons of Topt ranges to August habitat temperature metrics point to in-nature potential limitations for Hebgen nymphs due to cool temperatures in the summer at the Hebgen site and metabolic limitations for Varney nymphs due to summer maximum temperatures at the Varney site. August daily mean and minimum water temperatures measured at the Hebgen site usually remained cooler than the Topt90 range lower limit and never exceeded the Topt90 range upper limit. For the warmer Varney site, multiple temperature metrics showed August water temperatures to fall within the Topt90 range estimated for Varney nymphs, but August temperatures still exceeded the Varney nymph Topt90 range upper limit for nearly 100 hours or 1800 CDH. In fact, maximum August temperature measured at Varney was 22°C, which is 7°C above Topt90 and 3°C above the upper limit of the Topt90 range. As a result, nymphs at the Hebgen site had a relatively large thermal safety margin ranging from 5 to 7°C, depending on year and summary metric for maximum habitat temperature, compared to a thermal safety margin of −3 to 1°C at the Varney site. Short-term exposures exceeding the Topt range upper limit are unlikely to cause mass mortalities within the population, but sublethal effects related to periods of sub-peak (i.e. as occurring at ≥16°C for Varney nymphs) and moderately low (i.e. as occurring at ≥19.4°C for Varney nymphs) available AS to support work may impact long-term population dynamics of Varney salmonflies. Notably, nymphs at both Hebgen and Varney experienced temperatures within their Topt90 range for most days of August; therefore, during much of the summer, nymphs at both sites likely exist within their fundamental thermal niche, but the warmer Varney site regime likely warms to conditions at or exceeding the limits of the Varney nymph fundamental niche. Nymphs at the Varney site likely experience sublethal metabolic effects of warm temperatures, which could be the mechanism underlying previously reported reduced nymph density at the Varney site relative to at the Hebgen site. Although we show here that nymphs at the Varney site likely experience sublethal metabolic effects of warm temperatures consistent with population-level observations, it is important to note that we tracked metabolic responses of only one instar within a 4-year life cycle of salmonflies (Freilich, 1991; DeWalt and Stewart, 1995; Townsend and Pritchard, 1998). Investigation of temperature responses for other instar stages may reveal additional or stronger thermal regime constraints on salmonfly populations. We considered the potential that the threshold of 90% of peak AS we applied to estimate the Topt range was inappropriate by also comparing the Topt range threshold of 80% of peak AS, but trends for both Hebgen and Varney habitat temperatures relative to the optimal range remained similar regardless of threshold applied.

Comparisons of Topt ranges based on metabolic thermal responses to habitat temperature measurements support our previously published observations that populations of Madison River salmonfly nymphs experience limitations at warmer river locations downstream of the Hebgen site (e.g. at the Varney site) due to warm summer temperatures. Previously, our group and others observed salmonfly occurrence to be rare on the Madison River at locations where August weekly maximum temperatures exceeded 18.6°C (Huff *et al.*, 2006; Anderson *et al.*, 2019a). Additionally, Varney site salmonfly nymph body size was smaller, and abundance was ~70% of the abundance at the Hebgen site (Anderson *et al.*, 2019a; Table 1). Reduced body size of Varney relative to Hebgen nymphs likely reflect reduced growth or earlier initiation of mature tissues within Varney nymphs (Sweeney and Vannote, 1978). Consistent with our observations of a low-to-negative thermal safety margin at the Varney and relatively large thermal safety margin at the Hebgen site, the August 7DADMax temperature measurements for 2020 and 2021 exceeded the previously proposed limit of 18.6°C at the Varney, but not the Hebgen site.

Warmer thermal optima for Hebgen nymphs despite warmer habitat for Varney nymphs combined with our previously published observations of Varney site population limitations that correlate with warm habitat temperatures lead to the question: if nymphs can physiologically achieve warmer Hebgen-like thermal optima, why are Varney nymphs physiologically adjusted to a thermal optimal that appears to compromise performance within the Varney site thermal regime? We posit that the unexpected directionality of variation in MMR and AS responses across the two populations to warming could be a result of less seasonal and diel variation in water temperature experienced by nymphs at the Hebgen relative to the Varney site rather than August temperature patterns or extremes specifically. Other researchers have also argued to the importance of thermal variation to ectotherms (e.g. Morash *et al.*, 2021; Kefford *et al.*, 2022; Seebacher *et al.*, 2023). Due to the thermal stabilizing influences of the hypolimnetic Hebgen Dam discharge, Hebgen nymphs experience less thermal variation on diel and seasonal scales than Varney nymphs located 73 RKM downstream of Hebgen dam (Fig. 3E and F; Tables 2 and 3). As a result, across a typical year and a typical summer day, nymphs from the Varney site experience significantly lower (including sub-0°C in the winter) and higher temperatures relative to nymphs inhabiting the Hebgen site. Despite similar breadth of Topt for Hebgen (17.6–24.6° C = 7°C range) and Varney (11.4–19.4°C = 8°C range) nymphs, the temperature breadth over which nymphs maintained 90% of peak MMR was smaller for Hebgen (21.5–27.5°C = 6°C breadth) relative to Varney nymphs (11.9–26.3°C = 14.4°C breadth). Perhaps the larger temperature breadth for Varney MMR maintenance combined with a smaller peak MMR and reduced thermal sensitivity of RMR for Varney relative to Hebgen nymphs reflects the achievable limit of physiological optimizations to a wide diel range of temperatures that occur at the Varney site. The biochemical adjustments that allow for the high biological rates required (Huey and Kingsolver, 1989; Pörtner and Farrell, 2008) to flourish at the warmest water temperatures experienced within the Varney habitat may be sufficiently detrimental for nymph biology at the coolest temperatures that nymphs face a trade-off between biochemical optimization for the warmest and coolest temperatures experienced. This temperature difference can be a 10°C difference across a 24-hour period in the summer or >20°C difference between summer and winter at the Varney site (Tables 2 and 3). Although it is also possible that the sub-freezing temperatures of the Varney habitat directly kill nymphs, this trade-off postulation is consistent with our findings for both MMR and AS of a lower magnitude and cooler peak followed by a more gradual decline with warming above the peak in Varney nymphs relative to Hebgen nymphs (Fig. 4B). The narrower MMR Topt range and higher Topt for MMR and AS for Hebgen nymph may suggest that although Hebgen nymphs experience relatively cooler temperatures than Varney nymphs, due to the reduced thermal variation at the Hebgen site, Hebgen nymphs were better able to acclimate to warmer temperatures than the Varney nymphs and that thermal variation impaired biochemical adjustment in Varney nymphs to warm temperatures (Huey and Kingsolver, 1989; Pörtner and Farrell, 2008).

It is also important to consider other potential mechanisms underlying the unexpected directionality in Topt variation between Varney and Hebgen salmonfly nymphs. Potentially, due to behaviour and microhabitat selection, nymphs experience different thermal regimes than the ones we characterized. In this study, we characterized the thermal regime of surface waters, but the contributions of surface vs. hyporheic waters to the thermal regime experienced by salmonfly nymphs is not understood. Furthermore, we focused here on the August thermal regime as the warmest period experienced by nymphs; it is possible that nymph metabolic physiology is more responsive to the thermal regime at other points in the year. Additionally, we targeted the penultimate nymph instar of the salmonfly life cycle here because this is the life stage expected to accumulate the majority of the nutritional reserves that will fuel emergence into reproductive adults the following spring and the metabolic demands of warm summer temperatures that exceed optimal temperatures for aerobic capacity may impair accumulation of important nutritional reserves. Although the life stage targeted here has important implications to reproduction success and fitness, it is certainly possible that other stages of the salmonfly life cycle are more metabolically responsive to summer temperatures. Finally, other biotic or abiotic effects of habitat thermal regime on the ecosystem may be important mechanisms underlying the population trends previously observed between the two study sites. For example, the different thermal regimes may influence food availability or salmonflies may respond to the interaction between thermal regime, food availability and river flows (Albertson *et al.*, 2022). Additional research is needed to identify what temperature components most strongly regulate individuals and populations of salmonflies, whether their effects are experienced as lethal or sublethal in this system, how thermal responses across the life history of salmonflies vary and influence survival and reproduction and other biotic and abiotic effects of thermal regimes that may influence salmonfly populations.

Contrary to predictions that metabolic thermal responses of two salmonfly nymph populations would reflect the thermal gradient that defines their respective habitats, we report evidence of higher thermal optima for aerobic scope available for work in the cooler-habitat population relative to the warmer-habitat population. We suggest that diel, seasonal or both diel and seasonal thermal variation may more strongly influence the interpopulation variation in Topt than the mean or extreme temperatures experienced in the respective habitats. For example, the unexpected cooler thermal optima for the population inhabiting a warmer summer thermal regime may reflect biochemical trade-offs limiting simultaneous optimization to the warm and cool extremes occurring at diel and seasonal time scales. However, other mechanisms underlying the unexpected direction in inter-population variation observed here must also be considered. Both populations likely exist within their fundamental thermal niche, but the warmest summer temperatures experienced by nymphs originating from the warmer site potentially cause sublethal-to-lethal effects. Although these findings may explain previously reported reduced population abundance by 30% at Varney relative to the cooler site at Hebgen (Anderson *et al.*, 2019a), limitations during different seasons and life stages may also be important mechanisms underlying variation in the population along the river gradient. Regardless, our findings show that consideration of not just mean or maximum temperatures but also thermal regimes and thermal variation is critical for a comprehensive understanding of the drivers that govern intra-population aquatic insect responses to water temperatures. Our findings are some of the first to identify physiological requirements for multiple populations of salmonflies, which are an ecologically and culturally important resource in Western rivers. The unexpected metabolic responses to habitat thermal regime summer maximums highlight the need for continued research on the thermal metrics driving organismal performance in natural systems to better understand how aquatic ecosystem are responding to global change and to inform management actions to conserve the habitats that ecologically and economically valuable cold-water ectotherms depend upon.

## Supplementary Material

SupplementarySectionS3_coae043

IndivNymphData_Supplementary_coae043

## Data Availability

Data are provided in supplementary spreadsheet.
