# Supplementary material for: Thermal tolerance of giant salmonfly nymphs (Pteronarcys californica) varies across populations in a regulated river
Source: Conserv Physiol. 2024 Jul 5;12(1):coae043. doi: 10.1093/conphys/coae043 (PMC11225080; doi:10.1093/conphys/coae043)
Supplement: SupplementarySectionS3_coae043 [file supplementarysections3_coae043.pdf]

Supplementary Section

Table S1. Best-fit models estimating aerobic scope (AS) at temperature for salmonfly (*Pteronarcys californica*) nymphs from the Varney and Hebgen sites on the Madison River, MT using all considered allometric mass coefficients. Allo Coef: allometric coefficient adjusting mass; X: temperature; r2: adjusted r^2 of the linear model of fitted values versus observed values; Topt: optimal temperature, where AS peaked; Lower Topt90: the lowest temperature where AS remained within 90% of the peak AS; Upper Topt90: the warmest temperature where AS remained within 90% of the peak AS.

Population	Allo Coef	Model	r^2	Topt	Lower Topt90	Upper Topt90
Hebgen	0.00	$a+b\times X^2\times log(x)+c\times X^3$	0.44	21.3	17.6	24.6
Hebgen	0.10	exp(rho×X)-exp(rho×tmax-(tmax-X)/delta)	0.48	22.7	18.9	25.5
Hebgen	0.30	$a+ b\times X^2\times log(X)+c\times X^3$	0.54	20.9	17.1	24.2
Hebgen	0.60	$a+ b\times X^2\times log(X)+c\times X^3$	0.56	20.4	16.6	23.8
Hebgen	0.75	$a+ b\times X^2\times log(X)+c\times X^3$	0.50	20.2	16.3	23.6
Hebgen	0.90	$a+ b\times X^2\times log(X)+c\times X^3$	0.43	20.0	16.1	23.5
Hebgen	1.00	$a+ b\times X^2\times log(X)+c\times X^3$	0.37	19.9	15.9	23.3
Varney	0.00	$a+b\times log(X)^2+c\times log(X)+ d\times log(X)/X$	0.47	14.9	11.4	19.4
Varney	0.10	$a+b\times log(X)^2+c\times log(X)+ d\times log(X)/X$	0.52	14.9	11.4	19.5
Varney	0.30	$a+b\times log(X)^2+c\times log(X)+ d\times log(X)/X$	0.56	15.0	11.3	19.5
Varney	0.60	$a\times X\times(X- t0)\times(tmax-X)^{0.5}$	0.42	14.3	9.5	19.5
Varney	0.75	$a\times X\times(X- t0)\times(tmax-X)^{0.5}$	0.34	14.1	9.3	19.3
Varney	0.90	$a\times X\times(X- t0)\times(tmax-X)^{0.5}$	0.27	13.9	9.2	19.0
Varney	1.00	$a\times X\times(X- t0)\times(tmax-X)^{0.5}$	0.23	13.8	9.8	18.8

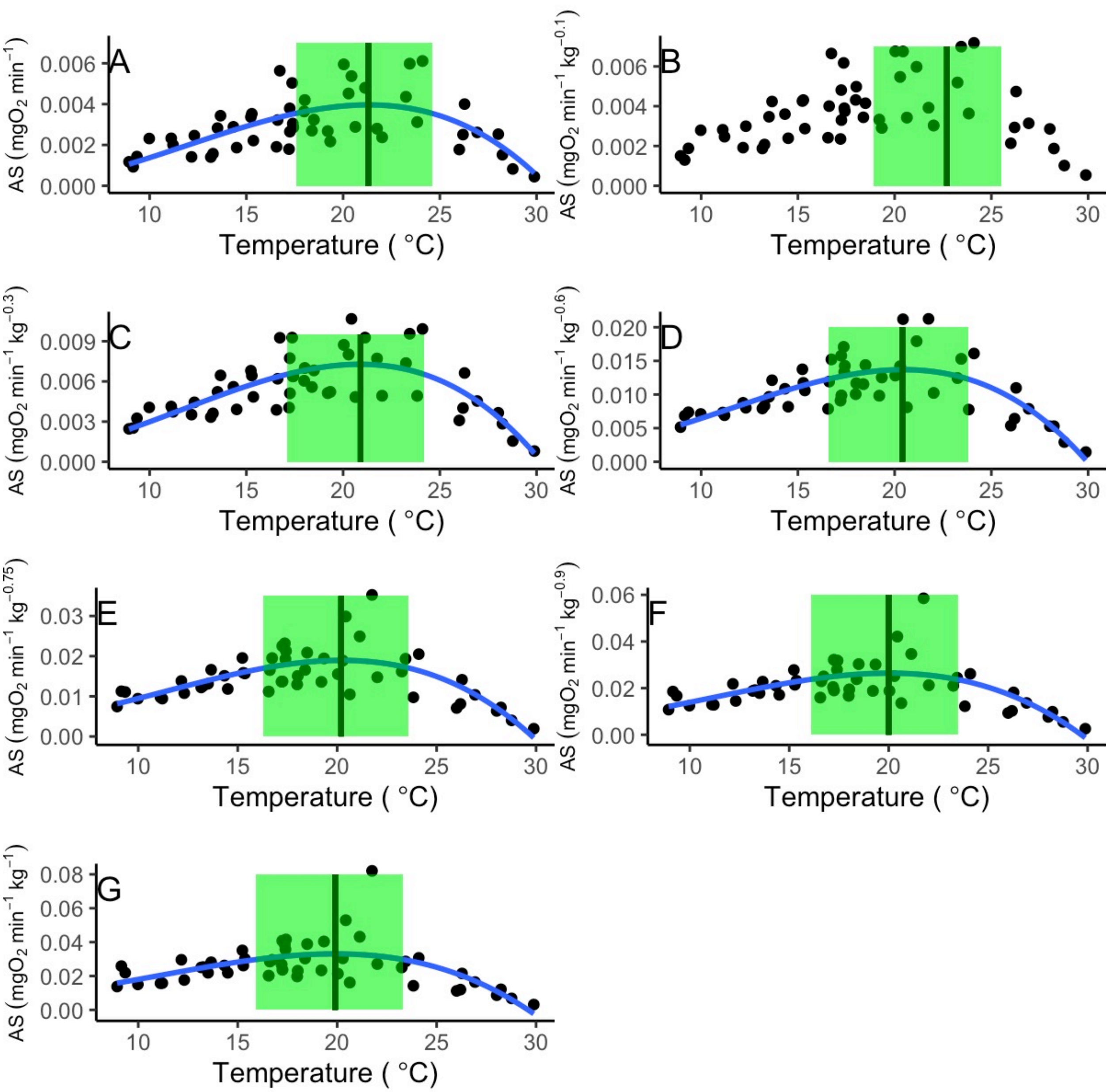

Figure S1. Fitted lined (blue solid line) of best-fit model estimating aerobic scope (AS) at temperature plotted over observed values (black dots) for salmonfly (*Pteronarcys californica*) nymphs from the Hebgen site on the Madison River, MT using all considered allometric mass coefficients. Aerobic scope was calculated by subtracting resting from maximum metabolic rate, both adjusted for nymph dry mass using allometric mass coefficients of A. 0.00, B. 0.10, C. 0.30, D. 0.60, E. 0.75, F. 0.90, and G. 1.00.

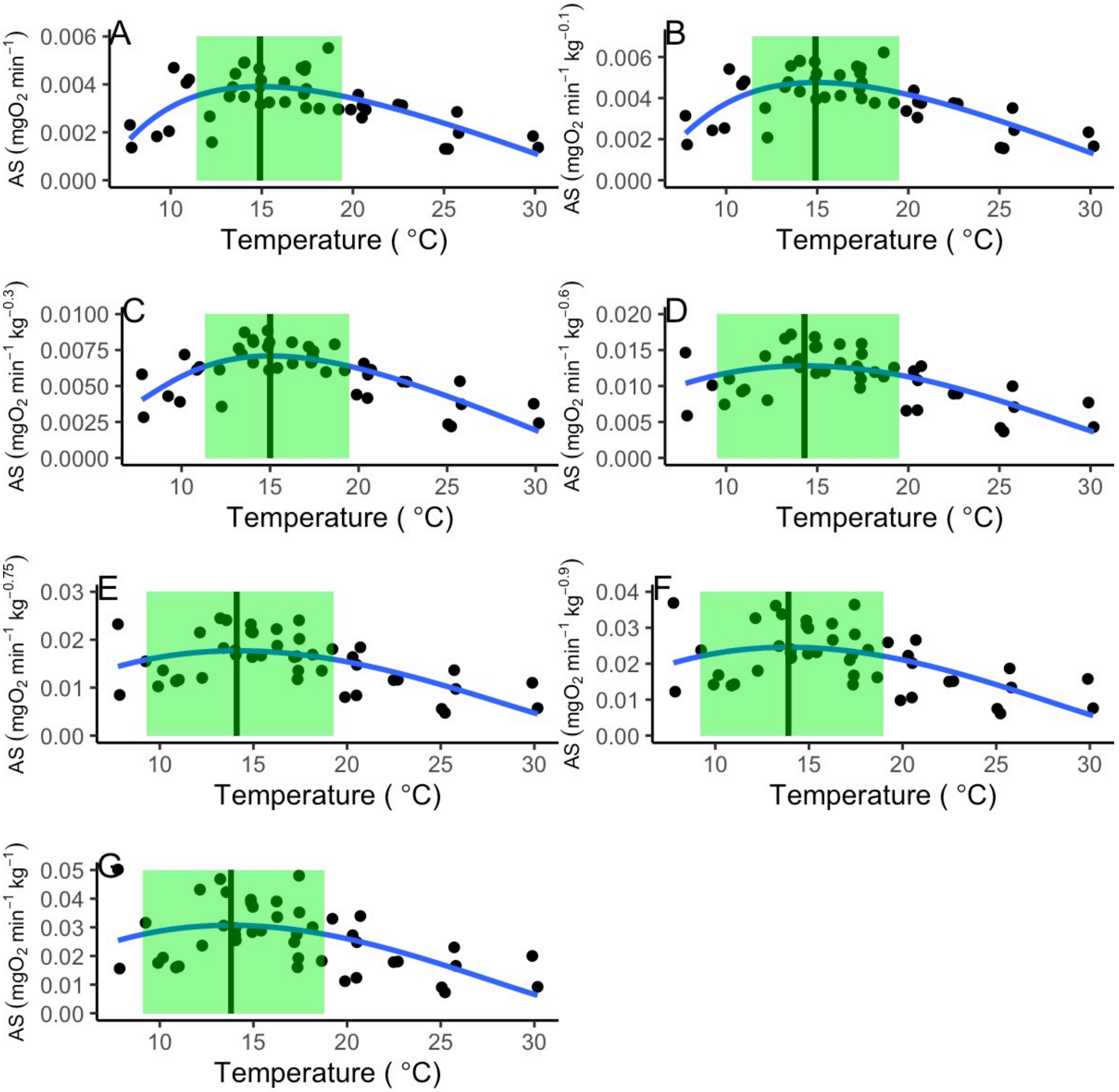

Figure S2. Fitted lined (blue solid line) of best-fit model estimating aerobic scope (AS) at temperature plotted over observed values (black dots) for salmonfly (*Pteronarcys californica*) nymphs from the Varney site on the Madison River, MT using all considered allometric mass coefficients. Aerobic scope was calculated by subtracting resting from maximum metabolic rate, both adjusted for nymph dry mass using allometric mass coefficients of A. 0.00, B. 0.10, C. 0.30, D. 0.60, E. 0.75, F. 0.90, and G. 1.00.

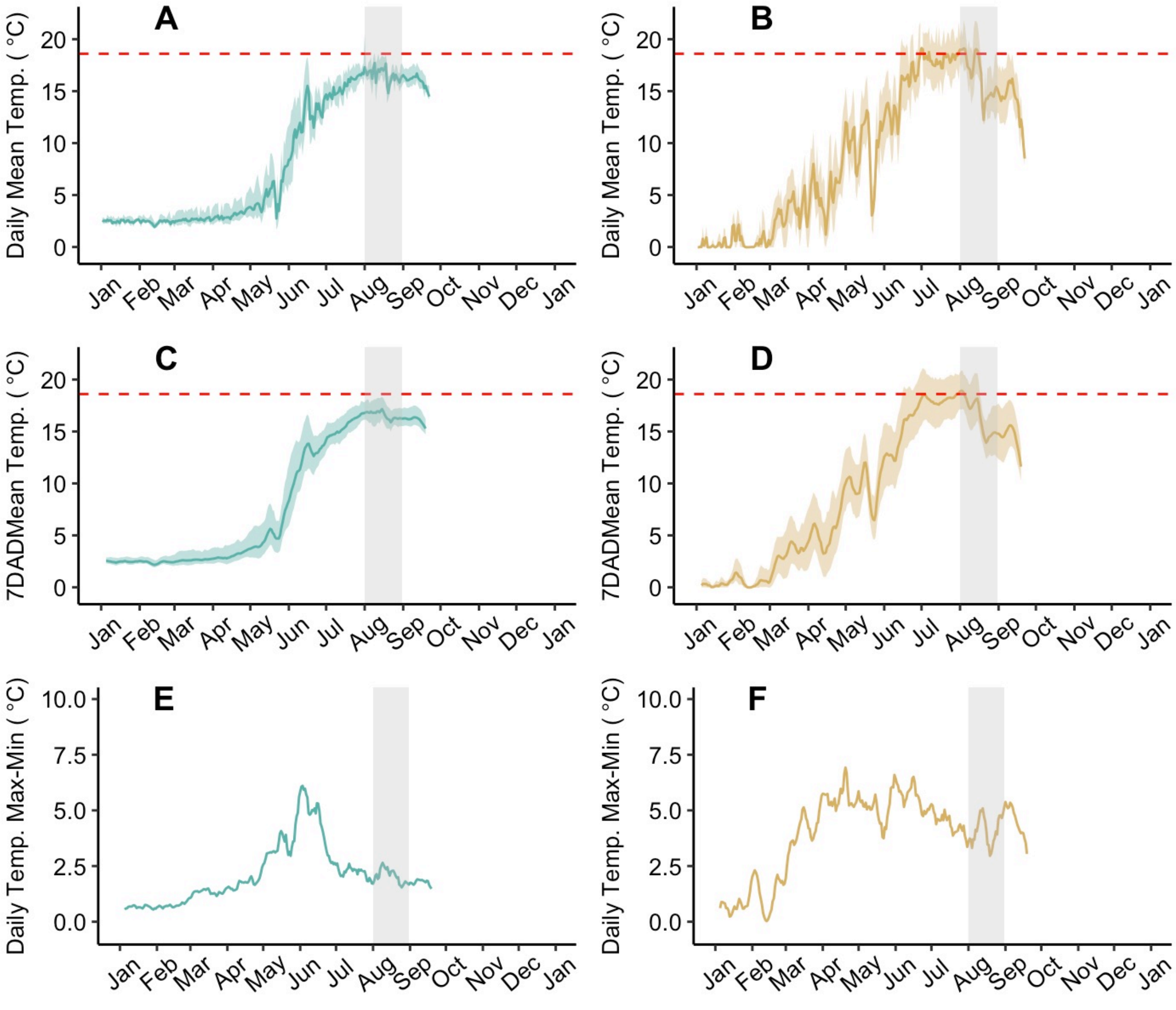

Figure S3. Temperature data logged in 2021 from two sites (Hebgen [green] in A, C, E and Varney Bridge [orange] in B, D, F) within the Madison River, MT where salmonfly (*Pteronarcys californica*) nymphs were collected for assessment of thermal responses for metabolism (see Fig 3 for 2020 temperature logs). A and B) Daily mean (solid line) and maximum and minimum (respectively shaded above and below the solid line) temperatures; C and D) sliding average of the seven-day average daily mean (7DADMean; (solid line)), maximum (7DADMax) and minimum (7DADMin) (respectively shaded above and below the solid line) temperatures; and E and F) diel variation (Daily Max-Min) calculated daily as the daily minimum recorded temperature subtracted by the daily maximum recorded temperature. Grey rectangles overlay August measurements. Salmonflies are relatively rare where August mean weekly maximum temperature exceeds 18.6°C (Huff et al., 2008; Anderson et al., 2019a), indicated by red dashed line in A to D.

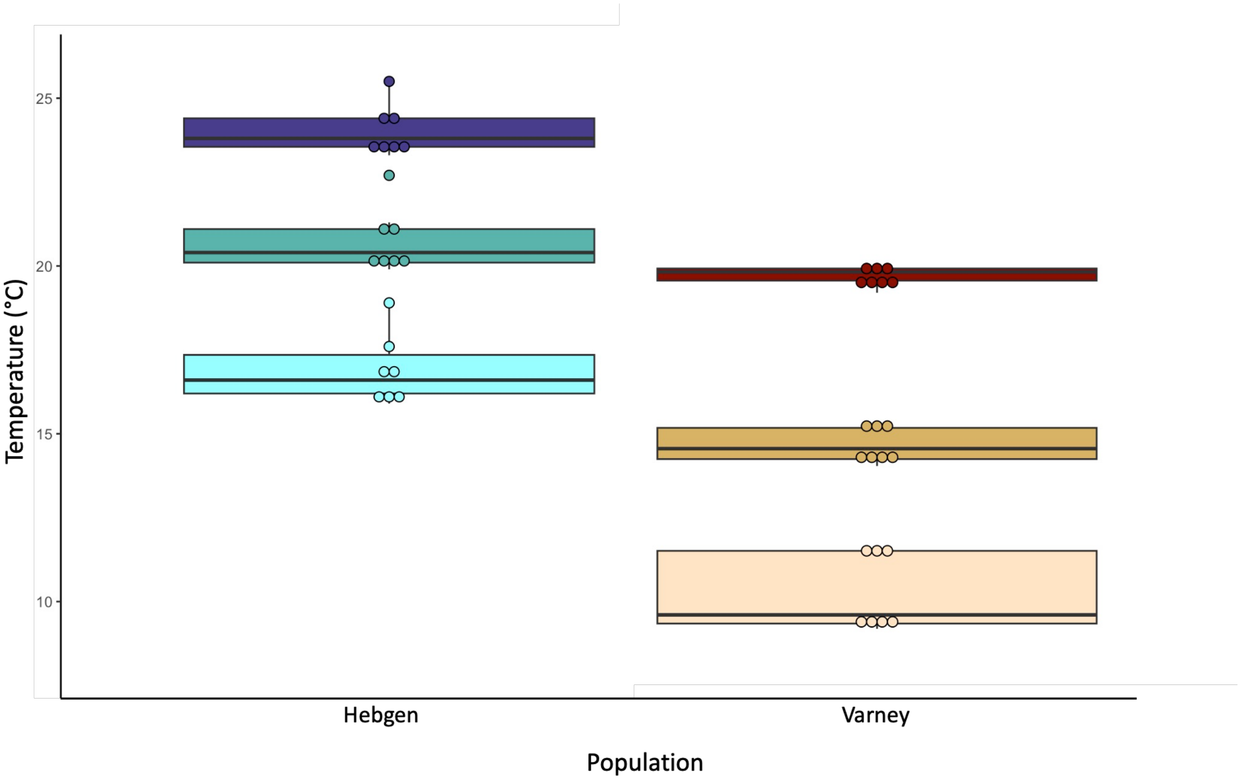

Figure S4. Optimal temperature (Topt; moderate shades), upper limit of the Topt range (darkest shades), and lower limit of the Topt range (lightest shades) estimates for Hebgen (blue-green hues) and Varney site (orange hues) nymphs based on aerobic scope responses to temperature calculated with resting and maximum metabolic rates adjusted for nymph dry mass using allometric mass coefficients (0.00, 0.10, 0.30, 0.60, 0.75, 0.90, and 1.00). Optimal temperatures were determined as the temperatures where aerobic scope peaked. Upper and lower limits of the Topt range were determined as the temperatures were aerobic scope first dropped below 90% of the peak aerobic scope as temperatures decreased (lower limit) or increased (upper limit) from Topt.
